# User and Provider Acceptability of Intermittent Screening and Treatment and Intermittent Preventive Treatment with Dihydroartemisinin-Piperaquine to Prevent Malaria in Pregnancy in Western Kenya

**DOI:** 10.1371/journal.pone.0150259

**Published:** 2016-03-17

**Authors:** Jenny Hill, Jenna Hoyt, Florence Achieng, Peter Ouma, Anne L’lanziva, Simon Kariuki, Meghna Desai, Jayne Webster

**Affiliations:** 1 Department of Clinical Sciences, Liverpool School of Tropical Medicine, Liverpool, United Kingdom; 2 Kenya Medical Research Institute/Centers for Disease Control Research and Public Health Collaboration, Kisumu, Kenya; 3 Centers for Disease Control and Prevention, Atlanta, Georgia, United States of America; 4 Disease Control Department, London School of Tropical Medicine and Hygiene, London, United Kingdom; Institut de Recherche pour le Développement, FRANCE

## Abstract

**Background:**

The World Health Organization recommends intermittent preventive treatment in pregnancy (IPTp) with sulphadoxine-pyrimethamine (SP) alongside long-lasting insecticide-treated nets (LLIN) and case management for reducing the risks associated with malaria in pregnancy in areas of moderate-to-high transmission in sub-Saharan Africa. Due to increasing *Plasmodium falciparum* resistance to SP, the search for alternative drugs or strategies to control malaria in pregnancy is a priority. We assessed the acceptability among pregnant women and health providers of intermittent screening and treatment (ISTp) and IPTp with dihydroartemisinin-piperaquine (DP) as alternative strategies in the context of an un-blinded clinical trial.

**Methods:**

Qualitative data were collected through ten focus group discussions with women participating in a randomized controlled trial to evaluate ISTp or IPTp with DP (multi-day regimen) versus IPTp with SP (single dose) in western Kenya. Individual in-depth interviews were conducted with 26 health providers working in the trial facilities and trial staff.

**Results:**

Women appreciated the advantages of being tested with a rapid diagnostic test (RDT) at every ANC visit (although a few women disliked finger pricks) and accepted that they would not receive any antimalarial when tested RDT-negative. There were differences in women’s experiences of the efficacy of antimalarials between the trial arms, with more women in the IPTp-SP arm reporting they had experienced malaria episodes. Side effects were experienced among women taking DP and SP. Although women and trial staff reported adherence to the full DP regimen within the trial, health providers were not confident that women would adhere to multi-day regimens in non-trial settings. Health providers recognized the advantages of ISTp in reducing unnecessary exposure to drugs, but lacked confidence in the reliability of RDTs compared to microscopy.

**Conclusions:**

Our findings indicate that, within a trial context, ISTp-DP and IPTp-DP were generally acceptable among both users and providers and were regarded as potentially valuable alternatives to IPTp-SP. Several challenges were identified the most important of which was concerns with achieving adherence to DP in non-trial settings, requiring operational feasibility studies in routine health systems. Policy adoption of ISTp with RDTs would require a major shift in thinking among health providers due to lack of confidence in RDTs.

## Background

Malaria infection in pregnancy is associated with severe maternal anemia, placental parasitaemia, low birth weight, and increased perinatal mortality. The World Health Organization (WHO) recommends intermittent preventive treatment in pregnancy (IPTp) with sulphadoxine-pyrimethamine (SP) at every scheduled ANC visit in the second and third trimester alongside long lasting insecticide-treated nets (LLIN) and case management for reducing the risks associated with malaria in pregnancy. Although SP currently appears to remain effective for IPTp in pregnant women [1] unlike in children, most likely because they have more immunity than young children [2], it will be important for countries to monitor the continued effectiveness of IPTp-SP in this population.

In the face of increasing *Plasmodium falciparum* resistance to SP [3] and reduced effectiveness of IPTp-SP in some settings [4, 5], the search for alternative drugs for IPTp and alternative prevention strategies to control malaria in pregnant women is a priority. A number of multicentre clinical trials of alternative drugs for IPTp and alternative strategies to prevent malaria in pregnancy have been conducted under the auspices of the Malaria in Pregnancy Consortium [6–8]. One potentially suitable alternative to SP for IPTp is dihydroartemisinin-piperaquine (DP) due to the long half-life of piperaquine and safety profile in pregnant women [9]. An alternative prevention strategy to the current policy of IPTp-SP being explored is ‘intermittent screening and treatment in pregnancy’ (ISTp), whereby women are tested for malaria parasites with rapid diagnostic tests (RDTs) at every scheduled ANC visit and those found to be positive are treated with an antimalarial [10]. Women who test negative therefore do not receive any antimalarial, unlike IPTp where antimalarials are given presumptively. Screening and treatment of pregnant women at first ANC visit (only) is practiced in some but not all health facilities in western Kenya, followed by IPTp-SP in subsequent visits in the second and third trimester.

A three-arm randomised controlled trial to compare ISTp or IPTp with DP versus current policy of IPTp-SP in an area of high SP resistance in western Kenya (ClinicalTrials.gov number NCT01669941) has recently been completed [9]. Nested within the main trial, we assessed the acceptability among pregnant women and health providers of ISTp-DP and IPTp-DP as alternatives to IPTp-SP. The findings will be used to inform implementation should either strategy be recommended for policy.

## Methods

### Ethics Statement

The study was approved by the ethical committees of the Kenya Medical Research Institute’s (KEMRI) National Ethics Review Committee, Kenya; the Liverpool School of Tropical Medicine, UK; the London School of Hygiene and Tropical Medicine, UK; and the Centres for Disease Control and Prevention, Atlanta, Georgia, USA. With participants’ prior agreement, verbal consent was obtained and recorded prior to the focus group discussions. In Kenya pregnant women aged 15–17 years are considered emancipated minors and were consented directly. All ethics committees and institutional review boards approved verbal informed consent to be obtained from FGD study participants and approved the study with the prior knowledge that minors aged between 15–18 years would be consenting for themselves. During transcription, any names were replaced with codes to ensure anonymity and digital recordings were deleted once transcription and translation were completed and quality approved.

### Study site and trial context

The qualitative study was conducted in the sites of the main trial in Siaya County, western Kenya, which was conducted between August 2012 and June 2014 [9]. Eligible women <32 weeks gestation attending for scheduled focused antenatal care (ANC) visits during their second and third trimester were randomly allocated to receive either: (1) IPTp with SP, (2) IPTp with a 3-day regimen of DP, or (3) scheduled screening with an RDT at every ANC visit and treatment of RDT-positive women with a 3-day regimen of DP. Women receiving IPTp-SP received a single dose of three tablets of SP taken under observation by trial staff. For women receiving DP for either IPTp or IST, the number of tablets administered was dependent upon the woman’s weight (range 2–4 tablets per dose). The first dose was administered under observation and the remaining two doses were taken home. A 5ml venous blood sample was collected for routine and study specific testing including haemoglobin levels, malaria (microscopy and polymerase chain reaction), HIV, and syphilis. All enrolled women received a long-lasting insecticide-treated net (LLIN) and were instructed on how to use them.

Trial participants were required to make 2–4 scheduled visits, depending on the gestational age at enrolment, at intervals of 4–8 weeks apart until delivery. Every fifth participant attending scheduled ANC visits was visited at home two days after their visit to assess drug safety, adherence and LLIN use. Trial participants were not responsible for any costs of ANC services received during the trial, including ANC registration, all drugs and tests, delivery and any in-patient care. They also received transportation reimbursement for all scheduled and un-scheduled ANC visits as well as free health insurance during their participation in the trial.

### Participants and study procedures

The acceptability study was conducted between July 2013 and April 2014, beginning 12 months after the clinical trial commenced. Focus group discussions (FGDs) were held with pregnant women enrolled in all three arms of the trial in two sites and in-depth interviews (IDIs) were conducted with trial staff and with health providers working in the four trial hospitals, but not involved directly in delivering trial interventions.

#### Focus group discussions with trial participants

**Fig 1:**
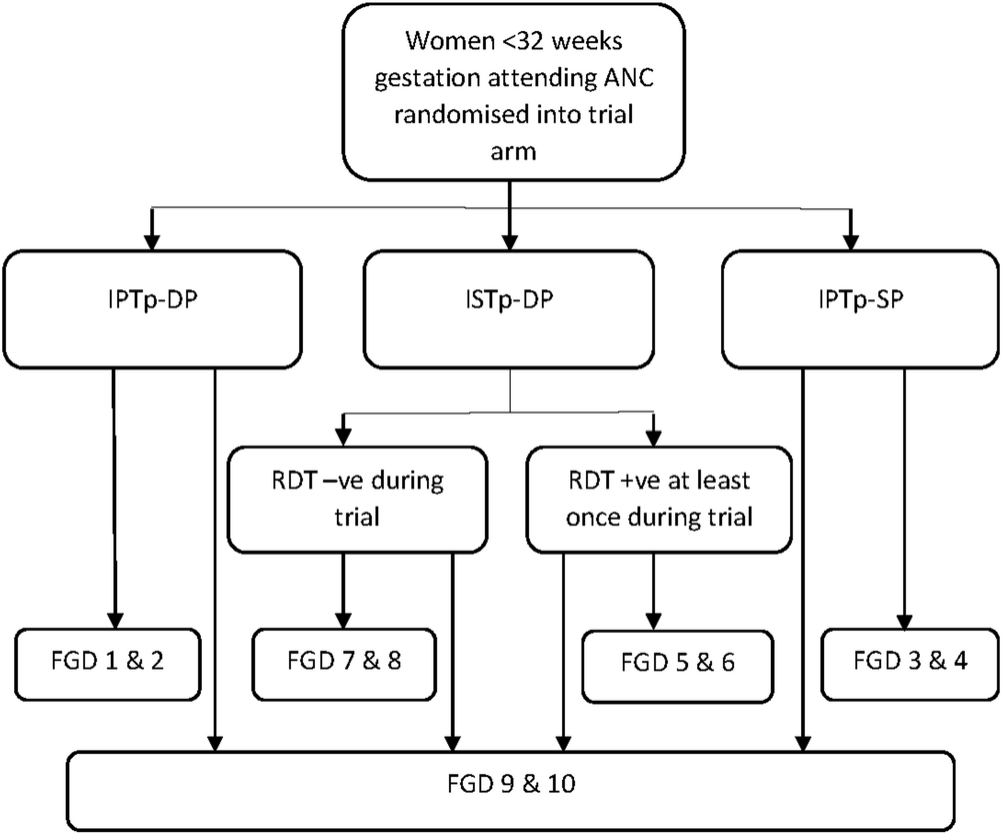
Allocation of trial participants to focus group discussion group by trial arm.

FGDs were undertaken with trial participants who had completed delivery in the catchment populations of Bondo County Hospital and Madiany sub-County Hospital. Ten FGDs were conducted with five groups of women purposively selected based on their trial intervention experience, namely women who received: 1) IPTp-DP; 2) ISTp-DP and who were RDT-negative, 3) ISTp-DP and who were RDT-positive at least once, 4) IPTp-SP or 5) a heterogeneous group receiving a mix of strategies (**Fig 1**). Two FGDs were conducted for each of these five groups, one in Bondo and one in Madiany. A total of 120 participants were identified through systematic random sampling from the trial database using a SAS random number generator to select 12 participants from each of the five intervention groups at each site. Women were contacted at home with the aid of community liaison staff employed by KEMRI, responsible for following up women at home, and invited to participate in an FGD held in a central location.

The FGD topic guides explored women’s experiences of ANC services received during the trial, perceptions of the services received, including blood and malaria tests, antimalarial drugs and regimens given in the clinic and to take home, and the factors which affected repeat attendance at ANC. The FGDs were conducted in the local language, Dholuo, by KEMRI’s social scientist (FA) and notes on verbal and non-verbal communication taken by a note-taker. FGDs lasted for approximately 50–90 minutes. All FGDs were digitally recorded and transcribed into English by FA within 48 hours of completion.

#### In-depth interviews with trial staff and MOH staff

In-depth interviews were conducted with trial staff and health facility staff from each of the four trial sites. Health facility staff were purposively selected and included the health facility in-charge, two nurses working in ANC clinic, one laboratory and one pharmacy staff from each facility, and a representative of the District Health Management Team from each of the four sub-counties. Two trial staff from each trial site were randomly selected.

One-to-one interviews with staff were conducted in English by FA using a semi-structured topic guide. Interview themes included: 1) perceptions of ISTp-DP and IPTp-DP versus IPTp-SP, 2) adaptations to working practices that would be required to implement ISTp-DP and IPTp-DP were they to become policy, 3) recommendations on factors to be considered to ensure effective implementation, and 4) perceptions of the feasibility of implementing ISTp-DP and IPTp-DP in the routine health system setting. Interviews were digitally recorded and transcribed by FA within 24–48 hours of completion.

### Data analysis

The FGD and IDI transcripts were entered into NVivo (QSR International) Version 10 for data management and analysis. Data from the FGDs and IDIs were coded separately by J Hoyt (JHo) and J Hill (JH) using a combination of pre-defined themes developed by two authors (JH and JW) based on the research questions that were developed into an analysis framework for each data set (FGDs and IDIs), and themes that emerged from the data using content analysis [11]. Any differences in coding were discussed until consensus was reached.

**Fig 2:**
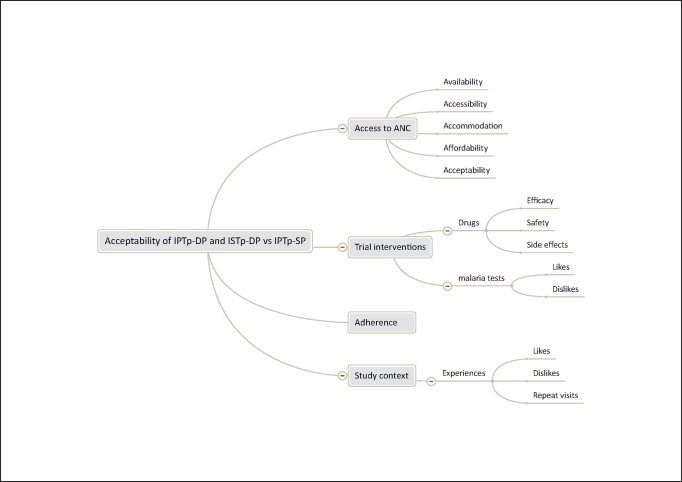
Analysis framework for focus group discussions with trial participants.

**Fig 3:**
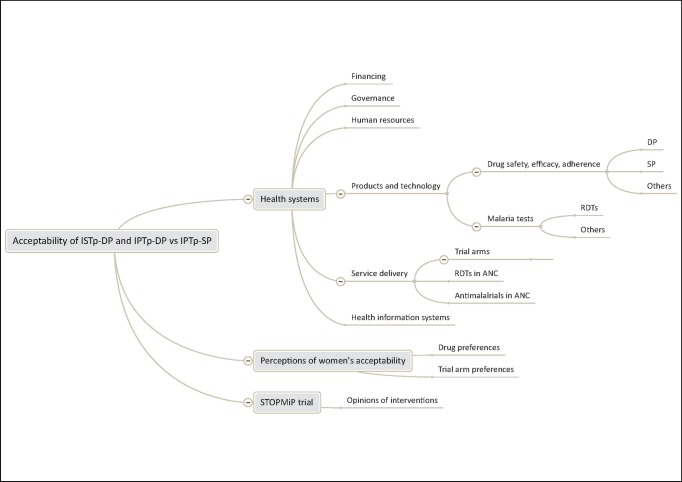
Analysis framework for in-depth interviews with health providers and trial staff.

The analysis framework for the FGDs comprised: treatment seeking behavior related to malaria in pregnancy (data not shown); access to ANC, as defined by Penchanksy [12] (availability, accessibility, affordability, accommodation, and acceptability); experiences and perceptions of ISTp-DP, IPTp-DP, IPTp-SP and other services received during the trial; adherence to MiP interventions; and perceptions and experiences of the clinical trial (**Fig 2**). The analysis framework for the in-depth interviews comprised: experience and opinions of the trial intervention arms according to the WHO health system building blocks [13]; perceptions of women’s acceptability of the trial interventions; experience, perceptions and opinions about the clinical trial (data not shown); and implications for implementation (**Fig 3**). During data analysis, all study participants were assigned anonymous codes. Women are identified by an individual respondent number, FGD number and code for the relevant arm of the trial; trial staff and health providers are identified by a respondent number, cadre and role within the health facility or trial.

## Results

A total of 61 women participated in the FGDs; their characteristics are provided in **Table 1**. The participants’ age range was 16–42 years, with an average across the FDG groups of 21–27 years. The majority of participants were married (n = 55; 90%) with between 1 and 9 children. Just over half the women had attained only a primary school education (n = 33; 54%). The most common occupations were business, farming and being a housewife.

**Table 1:** Characteristics of trial participants in the focus group discussions.

Site	Trial arm	FGD Group	No. of Participants (N = 61)	Average age (range)	Marital status (n)	Educational attainment (n)	Occupation (n)	Average no. of children (range)
Bondo	IPTp-DP	FGD 1	8	23 (17–26)	Married (7); Single (1)	Primary school Std 3, 7 (2); Secondary Form 1–4 (6)	housewife (4); business (4)	3 (1–5)
Madiany	IPTp-DP	FGD 2	8	26 (19–31)	Married (8)	Primary school Std 6–8 (6); Secondary Form 1–3 (2)	farmers (5); business (3)	3 (1–5)
Bondo	IPTp-SP	FGD 3	8	24 (20–28)	Married (7); Single (1)	Primary Std 8 (1); Secondary Form 2–4 (6); College (1)	business (6); teacher (1); housewife (1)	1 (1–2)
Madiany	IPTp-SP	FGD 4	5	27 (21–42)	Married (5)	Primary Std 7–8 (3); Secondary Form 2, 4 (2)	farmers (3); business (2)	3 (1–6)
Bondo	ISTp-DP positive	FGD 5	5	25 (19–36)	Married (5)	Primary Std 7–8 (4); Secondary Form 4 (1)	housewife (3); business (2)	2 (2–4)
Madiany	ISTp-DP positive	FGD 6	4	22 (19–24)	Married (3); Single (1)	Primary Std 7–8 (3); College (1)	business (2); teacher (1); farmer (1)	3 (1–5)
Bondo	ISTp-DP negative	FGD 7	6	25 (21–38)	Married (5); Single (1)	Primary Std 8 (3); Secondary Form 2 (1); College/ university (2)	student (2); business (1); farmer (1); housewife (1); community health worker (1)	3 (1–9)
Madiany	ISTp-DP negative	FGD 8	6	26 (19–38)	Married (5); Single (1)	Primary Std 7–8 (3); Secondary Form 3 (2); College (1)	student (1); business (2); farmer (3);	3 (1–6)
Bondo	Mixed	FGD 9	7	21 (16–30)	Married (6); Single (1)	Primary Std 5–8 (5); Secondary Form 2, 4 (2)	student (1); business (1); housewife (5);	3 (1–7)
Madiany	Mixed	FGD 10	4	22 (21–23)	Married (4)	Primary Std 8 (3); Secondary Form 3 (1)	business (1); farmer (2); housewife (1)	3 (2–3)

Abbreviations: DP, dihydroartemisinin-piperaquine; FGD, focus group discussion; ISTp, intermittent screening and treatment; SP, sulphadoxine-pyrimethamine; Std, Standard 1–8 (primary school)

In-depth interviews were conducted with 28 staff, including: 8 trial staff, 8 ANC staff, 4 laboratory technicians, 4 pharmacists and the District Medical Officer in each sub-county (**Table 2**). The main characteristics of the interviewees are provided in **Table 3**.

**Table 2:** Selection of trial staff and health providers for the in-depth interviews.

Cadre	Bondo District Hospital (N = 7)	Madiany sub-district Hospital (N = 7)	Lwak Mission Hospital (N = 7)	Siaya District Hospital (N = 7)	Sampling	Total (N = 28)
Trial staff in ANC	2	2	2	2	Random selection	8
Facility staff in ANC	2	2	2	2	Random selection	8
Lab technician in health facility	1	1	1	1	Only 1 staff in cadre	4
Pharmacist in health facility	1	1	1	1	Only 1 staff in cadre	4
District Medical Officer	1	1	1	1	Only 1 staff in cadre	4
**Study Arms**	IPTp-SP, IPTp-DP and ISTp-DP	IPTp-SP, IPTp-DP and ISTp-DP	IPTp-SP, IPTp-DP and ISTp-DP	IPTp-SP, IPTp-DP and ISTp-DP		

Abbreviations: DP, dihydroartemisinin-piperaquine; IPTp, intermittent preventive treatment; ISTp, intermittent screening and treatment; SP, sulphadoxine-pyrimethamine

**Table 3:** Health provider and trial staff characteristics.

Profession	IDI#	Highest professional qualification	Main tasks performed	Gender	Years in current facility	Role in ANC Y/N
Facility Nurse (2)	IDI 1	Diploma in Nursing	Works in MCH; family planning	Female	10	Yes (MCH)
Nursing Officer	IDI 2	Diploma in Community Nursing & Community Health	Oversees activities of other nurses	Female	3	Yes
Nurse	IDI 3	Kenya registered community health nurse	General nursing duties	Female	< 6 months	Yes
Nurse	IDI 4	Diploma in Nursing	In-patient department on maternity ward	Female	3	Yes (maternity)
Nursing Officer	IDI 5	Post graduate diploma in Health System Management	Oversees activities of others & general nursing duties	Female	7	Yes (MCH)
Nursing Manager	IDI 6	Diploma in Nursing	Coordinate activities in MCH dept	Female	6	Yes (MCH)
Facility Nurse	IDI 7	Enrolled Community Nurse	General nursing duties	Female	3	Yes
Nursing Manager	IDI 8	Degree in Nursing	Coordinate activities in MCH dept	Female	6	Yes (MCH)
Sister-In-charge	IDI 9	Diploma in Nursing	Coordinate regional HIV activities	Female	5	No
Medical Superintendant	IDI 10	Degree in Pharmacy	Medical Superintendent & in charge of Pharmacy	Male	7	No
Medical Superintendant	IDI 11	BSc in Medicine, BSc in Surgery	General supervision of entire health facility	Male	2	No
Medical Doctor	IDI 12	Medical Doctor	General coordination of activities in the health facility	Male	10	No
Lab Technician	IDI 13	Diploma in Laboratory Technology	Coordinates activities in the laboratory	Male	3	No
Lab Technician	IDI 14	Diploma in Medical Laboratory	Carries out testing of samples	Female	3	No
Lab Technician	IDI 15	Diploma in Medical Laboratory	Coordinates activities in the laboratory	Female	3	No
Lab Technician	IDI 16	Diploma in Medical Laboratory	Coordinates activities in the laboratory	Male	6	No
Pharmacist	IDI 17	Bachelor Degree in Pharmacy	Oversees procurement & dispensing of drugs	Male	2.3	No
Pharmacist	IDI 18	Higher Diploma in Pharmacy	Dispensing drugs	Male	10+	No
Pharmacist	IDI 19	Diploma in Pharmacy	Dispensing drugs	Male	3	No
Pharmacist	IDI 20	Diploma in Pharmacy	Dispensing drugs & managing inventory	Male	4	No
Nurse	IDI 21	Higher Diploma in Nursing/Community Health	Screening & enrollment of women in the study	Female	3	Yes
Nurse	IDI 22	Diploma in Nursing	Screening & enrollment of women in the study	Female	3	Yes
Site supervisor	IDI 23	Degree in Sociology	Supervise overall running of the study at this site	Female	1	Yes
Nurse	IDI 24	Registered Community Health Nurse	Screening & providing all ANC care	Female	n/a	yes
Study Coordinator	IDI 25	Registered Nurse	Supervise overall running of the study at this site	Female	6	Yes
Nurse	IDI 26	Diploma in Nursing	Screening, enrollment & collecting samples	Female	3	Yes
Nurse	IDI 27	Diploma in Nursing	Screening, enrollment & providing all ANC care	Female	2	Yes
Research Officer	IDI 28	Higher Diploma in Community Health & Development	Screening, enrollment & collecting samples	Female	5	Yes

Abbreviations: ANC, antenatal care; IDI, in-depth interview; MCH, Maternal and child health clinic

### Acceptability among trial participants

#### Experiences and perceptions of IST-DP, IPTp-DP and IPTp-SP

Issues affecting women’s acceptability of the trial interventions were categorized into four main themes: malaria tests, and the perceived benefits, side effects and adherence to the trial antimalarials, and trial arm (**Table 4**). Quotations are used selectively to illustrate only some viewpoints to reflect variability or consensus in the Results; a more comprehensive selection of quotations for each theme are provided in **S1 Table**.

**Table 4:** Experiences and perceptions on the acceptability of IPTp-DP or IST-DP vs IPTp-SP among trial participants, trial staff and health providers.

Themes	Pregnant women	Health providers/trial staff
	Sub-themes	Sub-themes
**Malaria tests**	Advantages of being tested every time; Understood that RDT-negative means no medication	Concerns about specificity and sensitivity of RDTs; RDTs could be useful in some settings (when no lab present); RDTs could reduce wait times for women & provide fast results; Multigravid RDT-negative women still expect antimalarial
**Benefits or efficacy of antimalarials**	Noticed difference in antimalarials given during trial vs previous pregnancies; Took [IPTp] SP but still got malaria; Believed the antimalarials had helped them & their babies	Concerns about SP resistance; Women taking DP in the trial had less malaria than those on SP; IPTp-SP has reduced the incidence of malaria in pregnancy
**Side effects of antimalarials**	Nausea & vomiting with DP; Nausea, vomiting & bad taste from SP; DP caused blackouts; Some “side effects” may be due to other conditions	DP caused unpleasant side effects in some women; SP caused side effects; SP has a high sulfa content that causes allergic reactions in some women
**Adherence to antimalarials**	Women took the antimalarials as prescribed in the trial; Complained that (adhering to) DP regimen was a ‘difficult’ experience	Concerns about adherence to DP as only first dose is given under DOT; Poor adherence to drugs given to take home; Lengthy dosing regimens decrease adherence; DP tablets are too big; Women prefer SP because you only take it for one day; Women stop taking meds when they start to feel better, or give medications to friends & family; Information needs to be given about importance of completing the dose; Information about timing of taking drugs must be easy for women to follow
**IPTp-DP or ISTp-DP vs IPTp-SP**	**ISTp:** Women were happy to know their malaria status at each ANC visit	**ISTp:** Good to treat those with malaria, reduce unnecessary medication; Concerns about the availability of RDTs and drugs (DP); Women who test negative don’t get drugs and they are not satisfied; Repeat screening requires women to make repeat ANC visits; Repeat screening may discourage some women from attending ANC. **IPTp-SP:** Concerns about SP resistance; Lack of RDT sensitivity means some women remain parasitaemic; need for hybrid strategy (ISTp and IPTp)

Abbreviations: DP, dihydroartemisinin-piperaquine; IPTp, intermittent preventive treatment; ISTp, intermittent screening and treatment; SP, sulphadoxine-pyrimethamine

**Malaria tests**: Many women saw the advantages of being tested for malaria at every ANC visit and not only at first ANC visit or when sick with malaria, although a few women disliked finger pricks. Women who tested RDT-negative appeared to accept that they did not receive any antimalarial, not even IPTp-SP. Two women in different arms spontaneously described an association between the absence of malaria in the blood, or a negative malaria test, and having taken antimalarials previously, one in the context of the trial (ISTp arm) and one in relation to treatment seeking for malaria episodes in the non-trial setting (IPTp-DP arm).

“R7: the study participants as I think have more advantages because the usual one when you go to the clinic and you are tested for malaria only that once for the first time [first ANC visit] and the rest you will not be tested and maybe you get malaria after you had been tested…therefore sometime you are asymptomatic to malaria and it eats up the baby from the womb but as for those enrolled in the study you are tested in each and every visit such that if there are chances that you acquire it afterwards then you can get treatment to prevent it…” IPTpDP FGD1

“R7: …I also like the method of blood testing because you may have some hidden diseases such as malaria, thereafter screening and testing is done and you being treated.” ISTpDP.N FGD8

**Antimalarials:** Women distinguished between the different trial drugs (antimalarials, folic acid, iron, vitamins) mostly by colour, name, type of intervention (IPTp and ISTp), and the number of times per day the drug was to be taken. Some multigravid women recognized that the antimalarials they received in the trial were different to those taken during their previous pregnancies. Many women reported that they were very happy with the antimalarials given because they believed they had helped them and their babies.

“R5: the drugs helped me and I never acquired malaria during pregnancy and even after I gave birth I had a safe delivery and the baby has not been sick up to this moment.” IPTpDP FGD1

Women’s perception of drug efficacy differed between the trial arms with a few women in the IPTp-SP arm reporting episodes of malaria whilst taking antimalarials, in one instance this was a woman who had vomited after taking SP.

“R3: what I like about these drugs [SP] is that they have prevented me from getting malaria…and until I gave birth I only had malaria once…in my own view they helped me because when I went the first time I took them and the second time I took them I vomited and then after that I was hit by malaria and so when I went back and they were changed now I used to take them and never vomited…and after this malaria disappeared…” IPTpSP FGD3

**Side effects**: The most commonly reported side effects from antimalarials were nausea and vomiting; other less frequently reported side effects included loss of appetite, blackouts, fever or body pains, weakness, and itching. When asked whether they thought that specific drugs were responsible for the side effects they were experiencing, many women insisted that they were caused by the antimalarials given in the trial. Women complained of side effects from both DP and SP. DP was associated with vomiting, nausea, loss of appetite, dizziness and blackouts, whereas SP was associated with nausea, vomiting and a bad taste in the mouth. It was acknowledged that some side effects, such as the blackouts reported by one woman, may have been due to other conditions experienced in pregnancy, such as ‘low blood levels’ (anaemia), and not DP as had been suggested. One woman in the IPTp-DP arm stated that she believed the side effects she experienced were due to not eating before taking the drugs.

“R3: because when you are pregnant you can experience a blackout and this sometimes may mean that the level of blood in your body is very low and the fact that the blood level is low also means that the two of you are sharing the blood.” IPTpDP FGD2

**Adherence**: Women reported taking most of the drugs given to them during their ANC visits, except for iron and folic acid, which many women said they disliked. They seemed content taking the antimalarials and respected the dosage and timing.

“R1: I swallowed…I was given nine. On the spot I swallowed three, the following day I swallowed three and the third day I swallowed three.” IPTpDP FGD1

A few women were hesitant and voiced concern about the DP regimen, as illustrated in the following quote, but the vast majority of women receiving DP for either ISTp or IPTp said that they adhered to the 3-day regimen.

“M: Respondent two, did you take the whole dosage?

R2: The other drugs that I was given I used to take because they told us that they will know when we don’t take the drugs except malaria drugs [DP] that I was not getting but it was difficult experience [meaning she was reluctant].” ISTpDP POS FGD5

#### Perceptions and experiences of ANC received during the trial

Women’s perceptions of the trial interventions were strongly influenced by the trial context. Women’s experiences of the ANC services received during the trial were said to be far superior to those received in previous pregnancies or by non-trial participants for a number of reasons, comprising four main themes: provider attitudes; provider professionalism; increased availability, quality and range of services; and free services and transportation. Quotations for each theme are provided in **S2 Table**.

### Acceptability among health providers and trial staff

#### Perceptions of IST-DP, IPTp-DP and IPTp-SP

Issues relevant to the acceptability of trial interventions among health providers were categorized into similar themes as for women: malaria tests; antimalarial efficacy, side effects and adherence; and trial arms (IST-DP, IPTp-DP and IPTp-SP). The themes and sub-themes emerging from health providers are compared to those from women in **Table 4**. Quotations for each theme are provided in **S1 Table**.

**Malaria Tests**: Across all cadres of health provider there was a strong preference for microscopy over RDTs as the ‘gold standard’ for malaria testing due to reliability of the results. Nevertheless, it was recognised that RDTs were useful in some settings, such as in health facilities with no labs or no power supply. RDTs were seen to have several benefits. The main benefits reported by ANC staff across all facilities was the reduction in time wasted by women queuing for lab results, and the results being available within minutes. A pharmacist and a nurse said this had two advantages, firstly, that women could see and have greater confidence in the result, and that treatment could be provided on the spot. The main drawback reported by two lab technicians and a facility in-charge was that RDTs are not specific or sensitive enough to produce reliable results.

“M: Now what would you think about the use of RDTs for diagnosis of malaria as an alternative of blood slides?

R: Okay. In my view the blood slide still remains the gold standard and in my view, a health facility like this one should and must continue using the slides for diagnosis….” IDI 10—Medical superintendant

“R: Okay, for the RDTs, they are there in case of power, electricity black out then they use RDTs, but it is being recommended that being that it is a District hospital, they should use the microscopic way of detecting, testing malaria.” IDI 6—Facility nurse

**Antimalarials (efficacy and side effects): DP:** Facility staff reported limited experience with administering DP to treat malaria in pregnancy. ANC nurses from two hospitals said DP was safe to use in pregnancy and was the preferred drug for the treatment of uncomplicated malaria in pregnancy, and a pharmacist said it was good for use in pregnant women given their low immunity. A facility in-charge had heard that DP offers longer protection against malaria and was therefore a good drug to use for prophylaxis. Several providers were awaiting the outcome of the trial before making any decisions about its efficacy. Two trial staff and one pharmacist witnessed some unpleasant side effects caused by DP in a few trial participants, specifically rashes, dizziness and nausea.

**SP:** Health providers reported routinely giving SP for IPTp and to women who complain of having malaria but test negative. Two pharmacists and a facility in-charge correctly stated that SP should not be given to women allergic to sulfa drugs and that it should be given with water to dilute the sulfa content, in line with policy.

**Adherence**: Several staff including three ANC nurses and two facility in-charges said adherence decreased for drugs with lengthy dosing regimens, citing quinine and DP. Considering pregnant women were also given paracetamol, multivitamins, iron and folate, and de-worming tablets, one ANC staff said women complained of drug ‘overload’. Other factors perceived by the providers to affect adherence were sharing drugs with family members, or stopping medication once women started to feel better. Most but not all trial staff thought that women adhered to the trial antimalarials. One was doubtful and another reported an instance where a trial participant was given trial antimalarials by her neighbour.

“R: So you see the empty blister packs and you are very happy that they took the drugs. But then later, there is a client who came to our clinic. And was telling us that she was feeling sick and was given some tablets by the neighbor, who is a study participant. And the tablets when we investigated were actually our DP. So probably she just removed them out of the blister packs and she kept them…. So when this neighbor was complaining that she was sick, then she was given. …So she took once and she felt worse. So she carried the drugs to the clinic … So when we checked [the drugs brought by the neighbour] they were actually our study drugs….There will be challenges but I think there will be challenges that can be avoided.” IDI 25—Trial staff

Three pharmacists said pregnant women needed very clear instructions on when to take drugs and explained they had no way of confirming whether women adhered to drug regimens. One medical superintendent noted that drug timing instructions needed to be clear so they were trying to discourage the ‘X times a day’ guidance currently given to clients and replace with specific intervals in hours. Two pharmacists said women should be advised that the dangers of a malaria relapse should they not complete the full regimen were far worse than any side effects they may experience.

**Trial arms**: **IPTp-SP:** Across all facilities, ANC nurses had the perception that IPTp-SP was effective in reducing malaria incidence when given under directly observed therapy (DOT). Several participants simply said it was a good strategy without elaborating as to why. One of the challenges to giving IPTp-SP by DOT was women wanting to take SP with food. Several nurses expressed concern about their perception of increasing resistance of the malaria parasite to SP, noting that some mothers taking IPTp-SP still got malaria.

“M: What do you think about the use of SP for IPTp in pregnant women? R: Mmm.., SP it was found that some mothers still get malaria. M: Even after using it? R: Even after using it we found that some of the mothers were still having malaria so I don’t know the feeling the people holding the study would have but that’s why they came with other drugs on trial.” IDI 7—Facility nurse

**IPTp-DP:** Perceptions of IPTp-DP among trial staff were that there was a lower incidence of malaria in this arm, with more women testing malaria negative at the monthly visits compared to the IPTp-SP arm. A medical superintendent commented that DP was safe to use in the 2^nd^ and 3^rd^ trimesters, gave longer acting protection, and would be a good alternative to SP, concluding that the trial would provide important evidence. Several trial staff however voiced concerns about adherence to DP since only the first dose is given by DOT.

“M: Did you ever hear the ANC staff employed by the government talking about the trial interventions? R: And sometimes when they also hear that we give it for three days. I have heard them asking questions that “will they take it” because I think in this society and environment people are not good at taking medications. Yeah, sometimes in the clinic, like SP you give it as DOT. You observe. Yeah, but if you give them the SP to go with home, some of them will not even take. So you would hear those raising questions but in all with time we continue talking to them and when it is a policy, I think all these will be looked at by the time it is put as a policy.” IDI 25—Trial staff

**ISTp-DP:** A clear benefit of ISTp reported by two ANC nurses, a medical superintendent and a pharmacist was that treating only active cases of malaria reduced unnecessary exposure to antimalarials in pregnancy, with infected women receiving appropriate treatment with an ACT as opposed to prophylaxis with SP. The medical superintendent also observed that ISTp was less likely to encourage the development of drug resistance. When asked what they thought about screening women at every ANC visit (as opposed to current policy, which is only at 1^st^ ANC visit) there were some practical concerns over the availability of RDTs, the cost and sustainability of scaling-up, and that the strategy relied on women making at least four ANC visits.

“M: How would you feel about every pregnant woman being given an RDT test at every ANC visit and if they are were found positive for malaria they would be given an anti-malarial drug. If they were negative they would not receive any anti-malarial? R: Yeah. When we get there it would be okay…..RDTs are expensive… And if they are available… that is a good intervention. Okay. We are also aware that the coverage of even the ANC attendance as we talk about people who have attended for four visits, we still have challenges. But on the other hand, if the RDTs were available, then we will protect the drugs which… we will protect the drugs we have from being misused because it is only those women who will have a positive RDT to be given the anti-malarial. But from the public health point of view, the expense that goes with a program like that needs to be given consideration to vis a vis the current practice. So if it is something that can be sustained, and the effect can impact positively towards the burden, alleviating the burden of malaria in pregnancy. Then it will be okay.” IDI 12- Medical superintendent

One trial staff remarked that women, particularly multigravida women, would need regular counselling about repeated testing at every ANC visit. Given the lack of reliability of the RDT results, the medical superintendent was concerned that RDT-negative women would not receive antimalarials, and observed that women with false positive tests would be given antimalarials even if they didn’t have parasites.

**ISTp vs IPTp:** When asked explicitly which strategy they preferred, IPTp or ISTp, health providers and trial staff often answered ambiguously. Although they accepted the idea of screening and treatment, most thought that prevention (IPTp) was also necessary. There was strong support across cadres, including trial staff, ANC nurses and one pharmacist, for continuing with prevention using IPTp.

“M: So out of the two we have IPTp and screening. In your opinion, which one do you think is better? R: Screening and IPTp? M: Yeah. R: I think all of them are better. We should screen and we should at least give prophylaxis. You know you can come and we give prophylaxis then maybe you are sick and I give you SP. It will not help you unless I screen and test you to know whether you have malaria, then I give you the drug which is going to help you.” IDI 4—Facility nurse

One trial staff commented that ISTp-DP was a good strategy because women who tested positive would be convinced of the need to take an antimalarial (DP), whereas with IPTp women do not always see the relevance of taking a drug if they did not have malaria (referring to IPTp-DP). One of the ANC nurses feared that regular testing for ISTp might deter women from repeat attendance at ANC.

“M: Based on your experience, what do you think is the best approach to be used in dealing with malaria in pregnancy? R: The best approach would be ISTp-DP, reason for me is that if you to test a mother and she turns positive, if you give her a drug she will take because she would be like I was tested and I had malaria she will have reason to tell, but you see if you give as in the IPTp-DP, a woman would not see the relevance. Somebody will say I’m not used taking drugs, I don’t even have malaria, but if you take someone positive, they will take the drug. However, if you just give, ISTp I think is the best one, it will help the compliance to be like 100 percent or even 90 percent. And also it would reduce the cost of resources.” IDI 26—Trial staff

#### Provider perceptions of women’s acceptability of the trial interventions

**Drug preferences**: Trial staff reported that pregnant women complained that the DP tablets were too big and some women broke the tablets in half. They reported that women noticed the difference in dosing regimens between DP and SP, with some complaining that taking tablets for three days was a burden.

“R: You know those who are taking DP, the person will rather go for SP. You know SP are only three tablets starting……., but DP you continue, so it always varies. There are those who feel like people who are in the SP, have lighter loads, and there are those who feel that those people who are in the DP are better because DP is a new drug.” IDI 26—Trial staff

Trial staff had mixed experiences of side effects reported by women, with one staff reporting no side effects from DP and one saying that no-one vomited with DP, whereas another said some women complained of dizziness and loss of appetite but despite these complaints had completed the regimen. Side effects from SP mentioned to the trial staff by women included nausea, vomiting and feeling ‘emaciated’.

**Trial arm preferences**: With regard strategy preference, trial staff said that some women preferred to take antimalarials only when sick. Conversely, ANC staff felt that IPTp-SP was generally well accepted for preventing malaria in pregnancy, as there had been a lot of counselling, such that if ISTp was introduced, RDT-negative multigravid women might need an explanation as to why they were not receiving an antimalarial.

“M: And how, how do you feel for those who just come and you screened them and unfortunately they are malaria negative, and yet they see others being given the drugs? What can you say about this? R: It has been a problem, some of them say that why is it that you give so and so drugs and you do not give me. M: I am going home without… R: Without any medication yet these are people that in other pregnancies they were given the three SP that used to be given.” IDI 21—Trial staff

#### Delivering interventions through the ANC platform

Overall, there was more support for ANC staff dispensing DP for either IPTp or ISTp than against, and this was reflected across all cadres and all health facilities including lab staff, pharmacists and ANC nurses (**S3 Table**). The main reason given in support of ANC staff dispensing antimalarials was that ANC would provide a one-stop-shop for the ANC package, thereby providing continuity of care and reducing wait times and queuing at the lab or pharmacy. However, one pharmacist thought ANC nurses would need to be overseen by a pharmacist to ensure correct practice and for stock management, given pharmacists are ultimately held responsible for any adverse effects if drugs are misused.

There was wide support for RDT testing to be performed in ANC clinics in terms of reduced wait times, faster results, and ensuring that all women are tested and can observe the results. It was noted that RDTs were low tech, easy to use, and could be easily integrated with HIV testing. Potential disadvantages cited were that regular testing could initially deter women from repeat ANC attendance, especially if malaria-RDTs were confused with HIV-tests, and that it would increase the workload of ANC staff. However, one ANC nurse noted that the benefits of using RDTs to screen women would outweigh the costs of an increased workload.

## Discussion

This is the first qualitative study to explore the acceptability among pregnant women and health providers of IPTp with DP, as a potential replacement drug for SP, and one of several studies that explores the acceptability of ISTp with either AL [14–17] or DP [18]. The study was conducted in the context of a clinical trial of IPTp or ISTp with DP vs IPTp-SP in western Kenya and, although not directly generalizable to a programme setting, provides important information to inform effective delivery were either of these interventions to become policy. Acceptability among women who participated in the trial centered on their views and experiences of malaria testing at every ANC visit, and the benefits, side effects and adherence to the antimalarials received in the trial arms. These views and experiences were heavily influenced by the trial context in comparison to services multigravid women received in previous pregnancies, or compared to non-trial participants attending the same ANC clinics. Although the health providers interviewed were not directly involved in the trial, many had a good understanding of the trial interventions and were aware of women’s experiences in the trial, and their views and opinions highlight important factors to consider for either strategy.

The majority of women who participated in the trial appreciated the beneficial effects of DP whether used for IPTp or ISTp, although there were complaints about the lengthy dosing regimen, a problem also identified in trials using AL for ISTp [17]. Several women experienced side effects to DP, though these were mild-to-moderate [19] and were similar to those reported by women in the SP arm. This mirrors tolerability results from the main trial, with nine documented episodes of vomiting after taking a study drug in all three trial arms (ISTp-DP -2 cases; IPTp-DP– 4 cases; and IPTp-SP -3 cases) [9]. This is in contrast to reports from women in a previous study where nausea and vomiting were more commonly associated with SP than ACTs [17]. The complaint of side effects occurring because drugs were taken without food was associated with both DP and SP, though clinical guidelines state that both drugs can be taken with or without food. The perception that SP must be taken with food has been identified in observational studies in non-trial settings [20, 21] and has been attributed to low adherence of IPTp provision by DOT [20, 22]. With regard to ISTp, women appreciated the benefits of malaria testing at every ANC visit instead of only at the first ANC visit, recognizing that some infections are asymptomatic yet harmful to the pregnancy. However, a few women were fearful of finger pricks. Adherence to DP during the trial as reported by women was ‘difficult’ due to the lengthy regimen and side effects, but they completed the regimen given the strong supportive environment of the trial. Health providers were skeptical that this level of adherence could be achieved in the routine ANC setting, as women are likely to stop taking drugs when they feel better. This notion is supported by a study in non-pregnant adults in a non-trial setting in Ghana, which found that adherence was strongest to drugs with minimal or no side effects, and perceived cure after the initial dose greatly affected adherence [23].

Health providers not involved in the trial reflected many of the views voiced by women. Facility-in-charges, nurses and pharmacists noted that adherence to the multi-day regimen and size of DP tablets poses a serious challenge to its future use for either ISTp or IPTp, although as one trial staff noted, adherence is likely to be higher among women who test positive in the ISTp strategy as these women will see test result and therefore the relevance of taking antimalarials. This obstacle was counterbalanced by the view that alternative drugs are needed in the face of growing resistance to SP. Trial staff were confident that women adhered to the DP regimen within the trial, but shared the concerns of health providers about replicating adherence in the routine ANC setting and noted that substantial counselling of women would be needed. The evidence on adherence to ACTs in the general population is weak, as found in a systematic review [24], and even less is known about adherence to ACTs during pregnancy [25], highlighting the need for better quality research to evaluate adherence to ACTs across diverse contexts using standardised methodologies.

Regarding ISTp, health providers from different cadres acknowledged its main advantage was limiting treatment to only active cases of malaria thereby reducing unnecessary exposure to antimalarials, but there were doubts about the reliability of RDTs, similar to studies in non-pregnant populations [26, 27]. The sensitivity of RDTs in pregnancy is lower than in non-pregnant populations [28, 29], and diminishes with increasing gestation (Feiko ter Kuile, personal communication). The broad skepticism across all cadres about the sensitivity or specificity of RDTs as a diagnostic method for malaria, and concerns that this strategy would leave some women unprotected, will need to be addressed should this strategy be recommended for policy. Overall, the unreliability of RDTs was seen as important as reducing wait times and speeding up the dispensing of treatment. Although RDT-negative trial participants appeared to accept not receiving any antimalarial, health providers saw this as a potential challenge among multigravid women who were used to receiving IPTp-SP. Importantly, health providers doubted that women would attend sufficient ANC visits to ensure monthly RDT-testing, and that ISTp could deter women who feared testing from coming to ANC. There was a strong preference for the continuation of IPTp across all cadres, even if ISTp were introduced as part of a hybrid strategy.

The acceptability of IPTp-DP and ISTp-DP among women and health providers was clearly influenced by the trial context, which raises some key issues to be considered when introducing either strategy into national programmes. Women in the trial appreciated the positive and helpful attitudes of the trial staff, the comprehensiveness, quality and availability of the care they received, and the fact that all services were free and transport reimbursement were provided, a finding consistent with the trial in Ghana [14]. These ANC experiences were in stark contrast to the services women received from routine ANC in their previous pregnancies. These factors will have directly encouraged women not only to return for their scheduled ANC visits, but also to accept aspects of care that they were not entirely comfortable with, such as regular testing and multi-day drug regimens. It remains to be seen whether this level of ANC attendance can be replicated and sustained in the non-trial setting, whether it is feasible to deliver either intervention through routine ANC, and whether women will adhere to DP for either ISTp or IPTp. A parallel study to determine the feasibility of implementing ISTp-DP vs IPTp-SP in routine health systems in western Kenya will help address some of these questions (to be published elsewhere), though additional feasibility studies will be needed for IPTp-DP. The cost effectiveness of ISTp vs IPTp and the cost of a policy change will also be published elsewhere.

### Study limitations

The experiences, perceptions and opinions of users and providers to the interventions delivered in a trial setting in one country are not generalizable to non-trial settings. The moderator and research team from KEMRI were likely to have been recognized by the participants as belonging to the same institution that conducted the trial, resulting in a social desirability bias. There were a limited number of women in some FGD groups, particularly in the ISTp groups (as shown in Table 1), which may have resulted in a narrower range of viewpoints.

## Conclusions

Our findings indicate that, within a trial context, IPTp-DP and ISTp-DP were generally acceptable among both users and providers and were perceived by health providers to be potentially valuable alternatives to IPTp-SP. Several challenges were identified, the most important of which was adherence to DP, highlighted by women as well as health providers and trial staff. As with SP, women will need appropriate information to promote acceptability and adherence to DP. Health providers in general lacked confidence in the reliability of RDTs. Consequently, any policy change from IPTp to ISTp only, i.e. not part of a hybrid strategy with IPTp, would require a major shift in thinking among health providers through targeted training and guidance on what should be done if providers suspect false negatives. Delivery of ISTp through ANC clinics will need to take into account workload and changes in staff responsibilities. As both interventions, particularly ISTp, will be more expensive to implement than IPTp-SP, the sustainability issues raised by health providers will need to be addressed.

## Supporting Information

S1 Table Women’s and health providers’ acceptability of IPTp-DP or IST-DP vs IPTp-SP. (DOC)

S2 Table Women’s experiences and perceptions of the trial context and impact on acceptability findings. (DOC)

S3 Table Health provider and trial staff perspectives in the context of the trial and implications for programmes. (DOC)

## Abbreviations

ANC: antenatal clinic
DOT: directly observed therapy
DP: dihydroartemisinin-piperaquine
IPTp: intermittent preventive treatment in pregnancy
SP: sulphadoxine-pyrimethamine
RDT: rapid diagnostic test
