# Supplementary material for: User and Provider Acceptability of Intermittent Screening and Treatment and Intermittent Preventive Treatment with Dihydroartemisinin-Piperaquine to Prevent Malaria in Pregnancy in Western Kenya
Source: PLoS One. 2016 Mar 17;11(3):e0150259. doi: 10.1371/journal.pone.0150259 (PMC4795545; doi:10.1371/journal.pone.0150259)
Supplement: S1 Table — (DOC) [file pone.0150259.s001.doc]

Table S1. Women’s and health providers’ acceptability of IPTp-DP or IST-DP vs IPTp-SP

| **Themes** | **Pregnant women**  **Sub-themes and quotations** | **Health providers/trial staff**  **Sub-themes and quotations** |
| --- | --- | --- |
| Malaria tests | **Advantages of being tested every time**  “R7: the study participants as I think have more advantages because the usual one when you go to the clinic and you are tested for malaria only that once for the first time and the rest you will not be tested and maybe you get malaria after you had been tested…therefore sometime you are asymptomatic to malaria and it eats up the baby from the womb but as for those enrolled in the study you are tested in each and every visit such that if there are chances that you acquire it afterwards then you can get treatment to prevent it (child crying)…” IPTpDP FGD1  “R6: I think someone not in the study maybe malaria was being tested for them when they went to be tested or when they felt that they are sick but ours was being tested anytime we went to the clinic.” ISTpDP.N FGD7 | **Concerns about specificity and sensitivity of RDTs**  “R: They can do the rapid diagnostic tests but the test should be confirmed with the microscope which is the golden standard. M: So is the rapid diagnostic test done by the nurses good? R: It is a good thing because for you to start treatment maybe like over the weekend, you do a quick reset…but it should be followed because the test has shown that with rapid test, maybe if somebody had been affected two weeks ago and had been treated, still it can be positive.” IDI 16 - Lab technician  **RDTs could be useful in some settings (when no lab present)**  “M: What do you think about the use of RDTs for diagnosis of malaria as an alternative to blood slides? R: I think it if more friendly. M: Mmm… R: It can be done at one spot and it can be done by the nursing aider, unlike the one for the microscopy that only requires the lab technologies. M: It is also quick. R: Yeah, it is also quick and can be used where there is no microscope.” IDI 8 – Nursing manager  **RDTs could reduce wait times for women & provide fast results**  “R: Yeah, it will make our mothers go home faster than wasting a lot of time in the line and in the lab and this might make them not to come another time because they took a lot of time the last time they were here.” IDI 7 - facility nurse  “M: What are your feelings about using RDT in routine ANC? R: It is nice because the provider who is seeing… the nurse seeing the client at the moment is able to test the client and get the results. So you are able to test by yourself and give the results.” IDI 8 – Nursing manager |
|  | **Understood that –ve RDT means no medication**  “R3: I was given medicine once because I was found with malaria only once.” ISTpDP.POS FGD5  “R6: when I first came, my blood was tested….M: Mmmmh (baby cries) and no malaria was found even other diseases….R6: Therefore I was given just normal drugs [meaning iron folate]…..I continue taking them. One day when I was feeling dizzy I decided to come back to the hospital. My blood was tested again and any drug by then I have not taken. What I was told is that maybe it is just some tiredness and some blood that they sometime taken from us is what can make can some time reduce therefore no disease was found in me and decided just to go back home.” ISTpDP.N FGD8 | **Multigravid RDT –ve women may still expect drugs**  “M: And how, how do you feel for those who just come and you screened them and unfortunately they are malaria negative, and yet they see others being given the drugs? What can you say about this? R: It has been a problem, some of them say that why is it that you give so and so drugs and you do not give me. M: I am going home without… R: Without any medication yet these are people that in other pregnancies they were given the three SP that used to be given.” IDI 21 - Trial staff |
| Benefits or efficacy of antimalarials | **Noticed difference in antimalarials given during trial vs previous pregnancies**  “R3: The difference that I saw from this my child whom I gave birth to under KEMRI/CDC while others I gave birth to them in our normal clinic is that the malaria medicine we got from the KEMRI/CDC are different from malaria drugs we got from these other normal clinics.” ISTpDP POS FGD5 | **Women taking DP in the trial had less malaria than those on SP**  “M: And what did you think about this trial? R: Okay personally in my own judgment I think that the drug, IPTp DP is very effective in the prevention of malaria in pregnancy because ever since we started the trial, I have not come across a client on this arm, IPTp DP who turns positive, malaria positive? M: Mmmm. R: So in my own judgment, IPTp DP is very good in preventing malaria.” IDI 24 - Trial staff  “R: Aaah that is a very broad question. One, I have realised that people who get aaah who are, who come down with malaria are in [ISTp] DP arm. I think because we do not give them intervention, we wait until they have malaria. For the SP, I believe that the perception is that SP is not functioning well, but I have realized that those mothers were given SP; they don’t come down with malaria as often as mothers in [ISTp] DP arm. That is what am seeing. Eeeh, but overall am not saying mothers in this arm are better off. They are more malaria negative than the others.” IDI 26 - Trial staff |
|  | **Took IPTp (SP) but still got malaria**  “R3: what I like about these drugs is that they have prevented me from getting malaria…and until I gave birth I only had malaria once…in my own view they helped me because when I went the first time I took them and the second time I took them I vomited and then after that I was hit by malaria and so when I went back and they were changed now I used to take them and never vomited…and after this malaria disappeared…” IPTpSP FGD3 | **Concerns about SP resistance**  “M: What do you think about the use of SP that is Fansidar for IPTp in pregnant women? R: Ok Fansidar there is resistance, some mothers if you give them quinine they still get malaria M: So what is your perception? R: There is resistance.” IDI 2 - nurse |
|  | **Believed the antimalarials had helped them & their babies**  “R5: the drugs helped me and I never acquired malaria during pregnancy and even after I gave birth I had a safe delivery and the baby has not been sick up to this moment.” IPTpDP FGD1  “R5: what I liked most about the drugs is because I took them and never had malaria until I gave birth…and this means that they prevented me from getting malaria…” IPTpSP FGD3 | **IPTp-SP has reduced the incidence of malaria in pregnancy**  “M: While I was taking you through the consent you heard me saying IPTP. Do you understand it? R: Yeah IPTP is Intermittent preventive treatment in pregnancy M: Yeah, do you see it? R: Ok it is a good programme. Yeah, because to me since it started here I’ve not seen any mothers coming with malaria conditions. So to me the numbers have reduced.” IDI 2 - nurse |
| Side effects of antimalarials | **Nausea & vomiting with DP**  “R9: when I swallow the DP drug I felt a lot of nausea… I had to sit down for sometimes after which I could then stand up.  M: now do you think…are you sure that all those problems you were seeing were because of these drugs? All that you have said, do you think?  R7: I think so because I started seeing all these only when I started using those drugs [meaning DP] and even during their visits I used to tell them all the problems that I was seeing…. I think it’s because of those drugs.” IPTpDP. FGD1 | **DP caused unpleasant side effects in some women**  “R: There was one mother who after taking the DP developed some rash. M: The, the skin rashes. R: She was withdrawn from taking the DP drug, so she just continued to be in the study but she never took DP until the end of our study. Then there is this one who had minor effects like nausea, vomiting after taking the drug… M: Mmm, Like headache. R: Yeah. Dizziness and slight headache.” IDI 21 - Trial staff |
|  | **DP caused blackouts**  “R5: personally the medicine that I was given….that…the yellow ones and the red ones I knew were drugs given to patients but the one that I did not know [meaning DP] was strange and I got scared and even was wondering how the drug would react with my body. Now it was the only drug that I feared and when I swallowed it I experienced blackouts and I lost appetite and sometimes after taking it I took around two hours just sleeping and then it brought about vomit. I vomited and experienced blackouts and even after drinking water I continually vomited and was wondering whether it was malaria or not…and therefore it was the only new drug that I swallowed and I had never swallowed it and it doing a lot of things in my body and therefore that’s the reason as to why I was scared about it.” IPTpDP FGD2 |  |
|  | **Nausea, vomiting & bad taste from SP**  “R1: for me when I take Fansidar, it brings a lot of saliva hence I feel like vomiting and some kind of foul taste in my mouth.”  “R2: for me I think it’s these drugs…Fansidar was responsible for the foul mouth but after some two days you are just fine.” IPTpSP FGD4 | **SP causes side effects**  “M: What are your feelings about giving IPTp? R: Some clients do complain that immediately we give them, we usually give them a DOT. We directly observe them taking the IPTp but most of the ladies say that immediately they take it here at the facility, they get the side effects, the nausea, the dizziness, so they prefer going with them IPTp back home but now the policy says we have to give them on DOT, we see them taking it ….yeah, observed.” IDI 6 - Facility Nurse  **SP has a high sulphur content that reacts with some women**  “R: Yeah, ok SP what I’ve been able to see was people complaining that some mothers react on the sulphur content in the SP; they get the black itchy patches. M: Mmh.” IDI 9 - Sister in Charge |
|  | **Some “side effects” may be due to other conditions**  “R3: because when you are pregnant you can experience a blackout and this sometimes may mean that the level of blood in your body is very low and the fact that the blood level is low also means that the two of you are sharing the blood.” IPTpDP FGD2 |  |
| Adherence to antimalarials | **Took the antimalarials as prescribed in the trial**  “R4: Antimalarial drug I took three tablets daily [DP], the yellow one [folic acid] I took two times daily and the one for blood [iron] I used to take twice daily.” ISTpDP POS FGD6  “R4: The white ones [DP] I took them all as illustrated one daily but the red [iron] and yellow ones [folic acid] I didn’t finish them. M: Why didn’t you take them all? R4: My heart dint like them.” ISTpDP POS FGD5  “R1: I swallowed…I was given nine. On the spot I swallowed three, the following day *…*I swallowed three and the third day I swallowed three.” IPTpDP FGD1  “R9: I was also given the same. I swallowed three on the spot and the following day I swallowed three…..next I swallowed three and the last they came to access how I was fairing *..”* IPTpDP FGD1 | **Concerns about adherence to DP as only first dose is given under DOT**  “…R: And sometimes when they also hear that we give it for three days. I have heard them asking questions that “will they take it” because I think in this society and environment people are not good at taking medications. Yeah, sometimes in the clinic, like SP you give it as DOT. You observe. Yeah, but if you give them the Sp to go with home, some of them will not even take So you would hear those raising questions but in all with time we continue talking to them and when it is a policy, I think all these will be looked at by the time it is put as a policy. Yeah.” IDI 25 - Trial staff  **Poor adherence to drugs given to take home (IPTpSP)**  “R: Initially we used to give them to go with them back at home and we used to see that most of them could come back with malaria slides being active, so it showed us that they were not taking them at home and that when they take at home they usually share the drugs among family members, they won’t take it a whole dose so that if we prefer, it is better if they take it when they are being seen.“ IDI 2 - Nurse |
|  | **Taking DP was a difficult experience**  “M: Respondent two, did you take the whole dosage? R2: The other drugs that I was given I used to take because they told us that they will know when we don’t take the drugs except malaria drugs [DP] that I was not getting but it was difficult experience.” ISTpDP POS FGD5 | **Lengthy dosing regimens decrease adherence**  “M: Now what do you think on the use of Quinine, AL, or Duocotexin [DP] for treatment of malaria cases in pregnant women? R: What I can say about the treatment of AL and Duocotexin [DP]. For AL, people do fear taking drugs and if you give them the twenty four tablets, you will hear some people taking it for even one day, and then they leave the remaining. They say that the drug is an overload but for Quinine, mostly we use Quinine IV.” IDI 4 - facility nurse  “R: I think very few people are faithful with drugs and will take them till they finish them, some of them, most of them when they feel well; they will stop taking the drugs, What about when they are not sick? (M and R laugh) there was an experience when I was working in Lwak, There was a client who went with the drug and as I told you, we pack them in the envelope and tell them this is for tomorrow and this is for the day after tomorrow and we give them the drugs. When the field assistants go home, they usually check the blister packs to ensure that the medicines were taken.” IDI 25 - Trial staff  **DP tablets are too big**  “M: What about the size, do you, do they have something to say about it? R: The size like, the DP is big, so they cut into two, they break it, for those who are saying it is big.” IDI 21 - Trial staff  **Women prefer SP because you only take it for one day**  “R: You know those who are taking DP, the person will rather go for SP. You know SP are only three tablets starting......., but DP you continue, so it always varies. There are those who feel like people who are in the SP, have lighter loads, and there are those who feel that those people who are in the DP are better because DP is a new drug.” IDI 26 - Trial staff  “M: Mgh. Did they have any questions about the drugs? R: Aaah… I think in every community you must find some people who will always give you some questions. We had a few who would complain and say sister, for three days. Some would wonder. Yeah. SP is always taken just for one day but this one for 3days so we had a few who would complain that way but I think the major work of a nurse is to re-assure the patients and tell them the importance of those drugs and how they are taken. After good explanation they would understand.” IDI 25 - Trial staff |
|  |  | **Women stop taking meds when they start to feel better, or give medications to friends & family**  “R: So you see the empty blister packs and you are very happy that they took the drugs. But then later, there is a client who came to our clinic. M: Mgh. R: And was telling us that she was feeling sick and was given some tablets by the neighbor, who is a study participant. M: Mgh. R: And the tablets when we investigated were actually our DP. So probably she just removed them out of the blister packs and she kept them. M: Exactly. R: So when this neighbor was complaining that she was sick, then she was given. M: Mgh. R: So she took once and she felt worse. So she carried the drugs to the clinic and she told us: yesterday I was sick and my neighbor gave me some drugs and say that “I even carried them, they are here”. So when we checked they were actually our study drugs. (M and R laugh). Yeah. There will be challenges but I think there will be challenges that can be avoided.” IDI 25 - Trial staff |
|  |  | **Information needs to be given about importance of completing the dose**  “M: The information you give about the drugs you dispense for malaria. R: The best information we always give is the special instructions, that’s the way to take them and how to take them then also other information that you need to give patients is that when they don’t take them at the right time required they might have a relapse or the treatment might fail so we always tell them the side effects of the drugs, the worse effects of the failure of the treatment when they are pregnant.” IDI 19 -Pharmacist |
|  |  | **Information about timing of taking drugs must be easy for women to follow**  “R: When it comes to Quinine, because it needs emphasis on the time, eight hourly, three times a day. So that eight hourly makes adherence more properly that when we say three times a day. M: Mmmm. R: And I know for the general population, they are used to three times a day and it is not proper adherence in the long run and the same also goes to AL. AL maybe …people generally know that it is twice a day. In the long run the general populations do not end up taking it the way it is required but it is supposed to be after every 12 hours. The beginning is a start close then you repeat after 8 hours. Again that is not strictly followed. And because of this timing I believe that there is going to be a lot of resistance most probably due to AL before the timing. It is really complex.” IDI 10 - Medical Superintendant |
| **ISTp** | **Women were happy to know their malaria status at each visit**  “R1: Apart from transport reimbursement, what encouraged me is that each time I visited the health facility I was tested for malaria and I always wanted to know whether am malaria positive or not.” ISTpDP POS FGD6  “R6: I think someone not in the study maybe malaria was being tested for them when they went to be tested or when they felt that they are sick but ours was being tested anytime we went to the clinic.” ISTpDP.N FGD7  “R7: the study participants as I think have more advantages because the usual one when you go to the clinic and you are tested for malaria only that once for the first time and the rest you will not be tested and maybe you get malaria after you had been tested…therefore sometime you are asymptomatic to malaria and it eats up the baby from the womb but as for those enrolled in the study you are tested in each and every visit such that if there are chances that you acquire it afterwards then you can get treatment to prevent it.” IPTpDP FGD1 | **Good to treat only those with malaria, reduce unnecessary medication**  “M: Okay. For us the project meant that, when women arrived at the ANC, they either get IPTp, or they get a blood test and if they are positive, they are given for treatment of malaria. Which of these approaches do you think is better? R: The approaches that you have said? M: Yeah. R: The best is to get tested then from the test if they are positive, they are treated, if they are negative, they are given IPTp. M: Why? R: Because you should not give a prophylaxis for someone who is already having the infection, instead you treat them.” IDI 8 – Nurse  “R: Yeah malaria has been there, but RDTs had been intensified some two months ago. Yeah, previously they were using other ways of treating the same malaria. Now that the RDTS are here; it assists in making detecting easier so that even a non-trained lab technician can do it. So with RDTs developed, it is okay if everybody is tested first because medicine is a poison and as we have said earlier it has side effects more so in pregnancy. So you should only give it if it is necessary by doing the rapid diagnostic test to see if it’s positive. If it is negative then we don’t give the drug then you save, and as you save that medicine it will be used as another …” IDI 18 – Pharmacist  **Concerns about the availability of RDTs and drugs (DP)**  “M: Which of these approaches do you think is better? The IPTp system or the blood. R: I would suggest the IPTp. M: Mmm, why? R: Because sometimes you can start the blood test and you run short of the RTDs. M: Mgh. R: And RDTs we have been having complaints of the erratic supply. So I would suggest that we continue with the prophylaxis of the IPTp.” IDI 6 - Facility nurse  “M: Mgh. Aaa… did you ever hear the ANC staff employed by the government talking about the trial interventions? R: About the trial interventions, yes I think aaah… severally some of them have asked me that; okay are you trying DP for IPTp that is Dihydroartemisin-Piperaquine for IPTp? And DP is even more expensive so even if it is put as a policy, would it really work? But there are times we even have stock outs of SP and we send patients to them…” IDI 25 – Trial staff  **Women who test negative don’t get drugs and they are not satisfied**  “M: And how, how do you feel for those who just come and you screened them and unfortunately they are malaria negative, and yet they see others being given the drugs? What can you say about this? R: It has been a problem, some of them say that why is it that you give so and so drugs and you do not give me. M: I am going home without… R: Without any medication yet these are people that in other pregnancies they were given the three SP that used to be given.” IDI 21 - Trial staff  **Repeat screening requires women to make repeat ANC visits**  “M: How would you feel about every pregnant woman being given an RDT test at every ANC visit and if they are were found positive for malaria they would be given an anti-malarial drug. If they were negative they would not receive any anti-malarial? R: Yeah. When we get there it would be okay…..RDTs are expensive… And if they are available… that is a good intervention. Okay. We are also aware that the coverage of even the ANC attendance as we talk about people who have attended for four visits, we still have challenges.” IDI 12- Medical superintendent  **Repeat screening may discourage some women from attending ANC**  “R: Issues about pricking and withdrawing blood from clients always carry several issues at the community level so when the client gets to know that every time you go to the clinic they have to be pricked, before they come to accept the practice a lot of them can even pull out of the MCH. They do not like the pricks but with time they get used to it and it will be business as usual just like HIV now, it is normal that the first visit and after every three months you have to be pricked for HIV if you go to the clinic.” IDI 5 - Facility nurse |
| **IPTp-SP** |  | **IPTp SP is failing, concerns about resistance**  “M: What do you think about the use of SP that is Fansidar for IPTp in pregnant women? R: Ok Fansidar there is resistance, some mothers if you give them quinine they still get malaria M: So what is your perception? R: There is resistance.” IDI 2 - nurse  “M: What do you think about the use of SP for IPTp in pregnant women? R: SP alone? M: Yeah. R: Mmmn, SP it was found that some mothers still get malaria. M: Even after using it? R: Even after using it we found that some of the mothers were still having malaria so I don’t know the feeling the people holding the study would have but that’s why they came with other drugs on trial.” IDI 7 - facility nurse  **Lack of RDT sensitivity means some women remain parasitaemic; need for hybrid strategy (ISTp and IPTp)**  “M: So out of the two we have IPTp and screening. In your opinion, which one do you think is better? R: Screening and IPTp? M: Yeah. R: I think all of them are better. We should screen and we should at least give prophylaxis. M: Eeh. R: You know you can come and we give prophylaxis then maybe you are sick and I give you SP. It will not help you unless I screen and test you to know whether you have malaria, then I give you the drug which is going to help you.” IDI 7 – Facility nurse  “M: So how would you feel about every pregnant woman requiring an RDT test at every ANC visit and if they are found positive for malaria, they would be given an anti-malarial drug? R: That is very okay, and another good thing should be prophylaxis. We should not always wait until they get malaria. So the better part is to prevent.” IDI – 17 Pharmacist |
