# Supplementary material for: User and Provider Acceptability of Intermittent Screening and Treatment and Intermittent Preventive Treatment with Dihydroartemisinin-Piperaquine to Prevent Malaria in Pregnancy in Western Kenya
Source: PLoS One. 2016 Mar 17;11(3):e0150259. doi: 10.1371/journal.pone.0150259 (PMC4795545; doi:10.1371/journal.pone.0150259)
Supplement: S2 Table — (DOC) [file pone.0150259.s002.doc]

Table S2. Women’s experiences and perceptions of the trial context and impact on acceptability findings

| **Analysis Framework** | **Themes and quotations** | **Health provider themes**  **(Table S3)** |
| --- | --- | --- |
| **Acceptability** | **Provider attitudes**  “R7: let me just say that with the first one [pregnancy] when I went to the clinic (child murmuring) they were not welcoming. It’s different from this one [pregnancy] in which they are very welcoming; everything else about them is good. They talk to you in a very open way until you feel free and open up for them to share every problem that you have but with the first one even if you have a problem you may end up going back home with it.” IPTpDP FGD1  “R1: what I saw is that when I went to the clinic they treated me well and they didn’t throw away my book like those other ones when I was late they would throw away your book and abuse you. The nurses at the clinic treated us well and talked nicely even if I was late and this made me have no stress going to the clinic….. What I didn’t like is that when we went to the clinic, they treated us well and even gave us sodas to take there but them when they came to our households to check on us, they were always in a hurry that they couldn’t even wait to have something.” ISTpDP POS FGD5  “R8: there was a time I went to the clinic and the way one of the pregnant women was handled instilled more fear in me and I swore not to come to the hospital. The nurse said “what kinds of things do you want to give birth to? Take that your swollen belly and lie here!”…and I was very much afraid that the sister will quarrel me the way she quarrelled the other one that’s what instilled fear in me.” IPTpDP FGD2  “R2: …in my opinion the nurses should not be quarrelling pregnant women. Sometimes you may also be sick and the kind of question she has asked you is a tough one… and also with the way pregnant women get upset easily…they ask the questions and when you don’t answer appropriately then they might end up abusing you “I did not impregnate you. You got the pregnancy out of your own wish”…this can also make people upset…” IPTpSP FGD3 | **Health workforce** |
|  | **Provider professionalism**  “R6…But something which impressed me, because I went for the visit when my days were almost due…..was that they told me “ if you hear of something unusual you come back or if you cannot come back you give us a call and we will come for you”. That was according to me very impressive because they worked with me professionally the way I feel they should be working...they offered me exclusive counselling and were there with me all the time, asking me how I woke up, sometimes they pay me visits and I noticed a very big difference because sometimes they may find you bedridden and they offer assistance to you…that according to me was very helpful.” IPTpDP FGD1  “R8: Personally I think we were given a lot of care and attention…and I still can remember a day that I came to the clinic and the other women in the next room also came to the clinic and the sister asked “ are they from this side or that other side?” and others replied that “ we are from that other side” and it was at the time the government health workers were on strike…and they were told that “if at all you are from that other side can you please get back home?”…and if you are from this side then you are most welcome and its at that point that I noticed some difference.” IPTpDP FGD2  “R9: On the day that I came to deliver I found a strike in place…I arrived in the hospital at night but when I called him [trial staff] he came very fast because I even found another woman giving birth at the door and now I was afraid and even wanted to go back home but they came so fast and this is what impressed me most” IPTpDP FGD1  “R7: it was different because even during the doctors strike when you went to the other hospitals you could not get the services but for us who are in the study it was just done the usual way. We got good services.” IPTpDP FGD1 |  |
| **Acceptability** | **Increased quality of services**  “R: a study participant is treated very differently from that one who is a non-participant (children crying)…the study participant are given quality treatment and any time you go you are asked questions about how you are faring but for the others who are not in the study, its taking your book for a test and to make the matters worse sometimes they keep you waiting for them when they are in their lunch break and sometimes you end up leaving without finishing everything.” IPTpDP FGD1  “R7: the study participants as I think have more advantages because the usual one when you go to the clinic and you are tested for malaria only that once for the first time and the rest you will not be tested and maybe you get malaria after you had been tested…therefore sometime you are asymptomatic to malaria and it eats up the baby from the womb but as for those enrolled in the study you are tested in each and every visit such that if there are chances that you acquire it afterwards then you can get treatment to prevent it.” IPTpDP FGD1  “R3: The blood tests and medicine given was done in a proper manner.” Mixed Group FGD10 | **Service delivery** |
|  | **Increased range of services**  “R7: I noticed a difference because, the first ones…this is my third born but with the first pregnancies your urine would be tested once during your first visit (children crying)…and not during the consecutive visits but with the current one you are given tests each and every visit. In every visit they test for the blood level, they test your urine and everything is done to you in each of all the visits but with the others after you have been tested in the first visit then nothing is done to you during the consecutive visits.” IPTpDP FGD1  “R2: I saw good things because as I had said earlier, I had a problem and they even took me for an X-ray (assume they mean ultrasound) and paid the bills for me but when my problem got too far I had to move further but they promised to be with me till i give birth. They treated me well unlike other clinics where they can abuse you till you give up.” ISTpDP POS FGD5 |  |
| **Availability** | **Increased availability of services**  “R5: we did not receive equal share of the services with the other women because the researchers were attending to us better than…”  “R7: it was different because even during the doctors strike when you went to the other hospitals you could not get the services but for us who are in the study it was just done the usual way. We got good services.” IPTpDP FGD1  “R3: while in this study, when you had started feeling the labour pain at home, you come and then the study will bail you out them, you don’t pay for the hospital bills M: eeh…number 1 R1: when you were about to deliver, you could call even if the place was too far, they could come to pick you to clinic.” IPTpSP FGD4 | **Service delivery** |
| **Accessibility/ Accommodation** | **Home visits**  “M:…so during the periods to which you used to come to clinic monthly visit, what really motivated you to be attending clinic?  R2: we had the morale because when you visited clinic [not clear] they could visit you at home and even they checked on how you lived.  R3: what really impressed me was the fact that they came to visit you at home even if they have nothing to tell you.” IPTpSP FGD4 | **Service delivery** |
| **Affordability** | **Free services and transportation**  “R8: I also come from very far but I used to try my best because the distance is too much and even if I was late I would comfortably lend someone and later return because whenever I went I would be given transport reimbursement…and this gave me a lot of encouragement to go to the clinic.” IPTpDP FGD 2  “R3: number three…I also got some very good help because on the day of delivery they took care of the hospital bill…they also gave me transport reimbursement whenever I went to and from the hospital. I also had a problem that required treatment…I was referred to Siaya and then from Siaya to Kisumu. They took care of the transport and the hospital bills for treatment and this therefore what I liked about them and this was different from how the rest were treated.” IPTpSP FGD3  “R2: …what I saw that was different was that they took care of all my expenses and even when I went for the ultra-sound they paid for the fee. Lastly when I went to give birth the nurses took good care of me compared to the other women who were non-participants…” IPTpSP FGD3  “R3: while in this study, when you had started feeling the labour pain at home, you come and then the study will bail you out them, you don’t pay for the hospital bills M: eeh…number 1 R1: when you were about to deliver, you could call even if the place was too far, they could come to pick you to clinic.” IPTpSP FGD4 | **Financing** |
