# Supplementary material for: User and Provider Acceptability of Intermittent Screening and Treatment and Intermittent Preventive Treatment with Dihydroartemisinin-Piperaquine to Prevent Malaria in Pregnancy in Western Kenya
Source: PLoS One. 2016 Mar 17;11(3):e0150259. doi: 10.1371/journal.pone.0150259 (PMC4795545; doi:10.1371/journal.pone.0150259)
Supplement: S3 Table — (DOC) [file pone.0150259.s003.doc]

**Table S3. Health provider and trial staff perspectives in the context of the trial** and implications for programmes

| **Analysis framework** | **Themes, sub-themes and quotations** |
| --- | --- |
| **Governance** | *Evidence for policy change*  **Fears about growing resistance to SP**  “M: What do you think about the use of SP for IPTp in pregnant women? R: SP alone? M: Yeah. R: Mmmn, SP it was found that some mothers still get malaria. M: Even after using it? R: Even after using it we found that some of the mothers were still having malaria so I don’t know the feeling the people holding the study would have but that’s why they came with other drugs on trial.” IDI 4- facility nurse |
| **Financing** | *Increased cost*  **DP is more expensive than SP**  “M: Mgh. Aaa… did you ever hear the ANC staff employed by the government talking about the trial interventions? R: About the trial interventions, yes i think aaah… severally some of them have asked me that; okay are you trying DP for IPTp that is Dihydroartemisin-Piperaquine for IPTp? And DP is even more expensive so even if it is put as a policy, would it really work? But there are times we even have stock outs of SP and we send patients to them. M: Mgh. R: And sometimes when they also hear that we give it for three days. I have heard them asking questions that “will they take it” because I think in this society and environment people are not good at taking medications. Yeah, sometimes in the clinic, like SP you give it as DOT. You observe. Yeah,but if you give them the SP to go with home, some of them will not even take So you would hear those raising questions but in all with time we continue talking to them and when it is a policy, I think all these will be looked at by the time it is put as a policy. Yeah.” IDI 25 - Trial staff  *Sustainability*  **Erratic supply of RDTs could have negative implications for IST**  “R: Because sometimes you can start the blood test and you run short of the RTDs. M: Mgh. R: And RDTs we have been having complaints of the erratic supply. So I would suggest that we continue with the prophylaxis of the IPTp.” IDI 6- facility nurse |
| **Human resources** | *Workload*  **MCH is understaffed, could have negative effect on IST**  “R: And not KEMRI/CDC. I would like to talk on their behalf. M: Mgh. R: KEMRI/CDC is a bit well staffed, but MCH is very poorly staffed. M: Mgh. R: This strategy is going to be effective I we improve on the staffing. Because why I am saying that is, it is very tough to have a close bond and friendship when you are so busy. You have like a thousand patients waiting for you outside. You just want to work and clear the queue. M: Mmmm, Exactly. R: I am saying this from experience because there are times we have gone to help in the MCH facility when they are so short staffed.” IDI 25 - Trial staff  “M: What about work load? Won’t it be an added load to what you do? R: Yeah. I accept that it can be and given that we are very few in the facility. M: So, how would you deal with this? R: Okay. For me, my suggestion is that I would let the lab to continue doing it given that we have much of work load in the ANC.” IDI 6 - facility nurse  **STOPMIP trial affected workload – increasing for some and decreasing for others**  “M: Concerning your work now as the lab technician. R: Okay, my work in the laboratory. M: Yes. R: With ANC? M: Yes, with ANC. R: It is okay, because I do not feel over loaded. STOPMiP brought staff here, so I think it is not over burdening.” IDI 13 - Lab technician  “M: Work load like in terms of… Okay what you are doing versus the number of people doing it? R: Like now in the ANC, the lady who used to assist us in doing the PMCTC is the lady from STOPMIP and she is currently on leave, so you see I am forced to double deal my duties.” IDI 3 - facility nurse  **IST would increase ANC staff workload but benefits would be worth it**  “M: Mgh. So like you have said and I think if I can remember well, when I talked of the RDTs that they are tested there, It is convenient because they see the results at that very point and therefore we do not need to have a lab technician, how do you feel about the work load? Compare it… as now, then you are added at another task of screening these mothers, maybe giving them drugs at the same time how do you feel about that, the work load, maybe compared to the number of staffs you have. R: Of course it appears like an extra work load. Yeah, but I think if you can detect malaria earlier in a pregnant woman, it is better than waiting to treat. M: Mmm…, the work load vis a vis the convenience to the client, yeah. R: The benefit outweighs the argument of the work load. M: Mgh. R: Yeah, I do agree it will be an added task.” IDI 1 - facility nurse  **Role change/sharing**  “M: And therefore how will you feel if the pregnant women attending ANC who tested positive for malaria were given their anti malarial drug treatment by the ANC staff? R: I would feel good but it would be okay when they consulted with my office and we discussed about it, and when we’ve now sat down and discussed it we give the way to do it or the way forward, and we have somebody from my office doing the regular visits and checking or supervising what they are doing so it will be comfortable but when they are doing it on their own I won’t be comfortable that much.” IDI 19 – pharmacist  “R:…As for as for the drug utilization, we give if the protocols are used because protocols of drug management says that any medicine that comes through this hospital should pass through the drug store and then from the drug store it is documented M: It must be… R: If protocol is to be observed, the pharmacist will use this to order it from the store then it comes here. When it has come here then nurse at the ANC will come here to get it, so if I’ve given 10 doses of fansidar to her, then she comes after a week to get another 10 doses, I will know that she has given it there, in other words I can do reconsumption even if am not there giving from this angle…and then monitor.” IDI - 18 Pharmacist |
| **Products and technology** | **Microscopy is better than RDTs**  “M: Okay. And what do you think about the use of RDTs for diagnosis of malaria as an alternative to blood slides? R: Blood slides remain the best. M: Okay. So would you recommend patients to go for the blood slides rather than the RDTs? R: Exactly.” IDI 13 - Lab technician  **RDTs are not sensitive or reliable enough for diagnostics**  “R: They can do the rapid diagnostic tests but the test should be confirmed with the microscope which is the golden standard. M: So is the rapid diagnostic test done by the nurses good? R: It is a good thing because for you to start treatment maybe like over the weekend, you do a quick reset…but it should be followed because the test has shown that with rapid test, maybe if somebody had been affected two weeks ago and had been treated, still it can be positive.” IDI 16 - Lab technician  **RDTs have some benefits in low resources settings**  “M: Ok and between RDT and other methods of testing which one do you prefer most? R: Ok RDT is good especially in cases where there is no laboratory, technician or chemologist because anybody can do it after training compared to the others where we need a microscope and this one you are being trained.” IDI 2 - nurse  **Women can see the results themselves with RDT**  “M: Yeah so you show the patient after doing the result? R: After doing the result and in fact you can even give the patients to see how its running now the patient can be sure that they have malaria, it will provide more confidence to the patients in that matter than the slides that you just come up with a result, you cant tell a patient to see the slides if they are positive or negative but with the RDTs I think they are the best.” IDI 19 - Pharmacist |
|  | **DP is a good, safe drug to use in pregnancy**  “M: What do you think is the best drug to give a pregnant woman to treat malaria and why is it the best now? R: The best now is Duocotexin. M: Mmm. R: Yeah, it is the best drug we are using, but I just think due to the research done and it was found to be better for pregnant women.” IDI 4 - facility nurse.  **DP offers longer protection against malaria**  “M: Have you heard any comments from the mid-wives or nurse on giving DHA-Piperaquine to pregnant women? R: No, so far I have not heard anything about that, but I have heard it in studies being carried out in other places that DP provides longer protection against malaria. That is what I have heard. Pro… what is it called...? Prophylaxis. It can provide longer protection against malaria because it has more …it can stay in the system for some period of time before it is totally eliminated in the system so that it may be one of the advantages and something I have heard from somewhere else but not really from the mid wives or something.” IDI 10 - Medical Superintendant  **DP has some unpleasant side effects**  “R: There was one mother who after taking the DP developed some rash. M: The, the skin rashes. R: She was withdrawn from taking the DP drug, so she just continued to be in the study but she never took DP until the end of our study. Then there is this one who had minor effects like nausea, vomiting after taking the drug… M: Mmm, Like headache. R: Yeah. Dizziness and slight headache.” IDI 21 - Trial staff  “R: There is no facility apart from those that are running the studies that give the DP as you call it. It is not supplied anywhere in the facilities. M: okay. R: That’s why I am telling you it is not going to be hard on this since DP that they are giving there, (names the study clinician) always calls me when there is something coming up seriously. M: Mmm. R: Of the rashes. We do manage the rashes.” IDI 17 - Pharmacist  “R: Not dizzy, they feel kind of tired, and then nausea is also another side effect. Someone would tell you after that after she took this drug, she felt much emaciated and the same SP... When they take DP, they tell you that they feel emaciated.” IDI 26 - Trial staff |
| **Service delivery** | **RDTs in ANC**  **Reduces wait times for women at ANC**  “R: Yeah, it will make our mothers go home faster than wasting a lot of time in the line and in the lab and this might make them not to come another time because they took a lot of time the last time they were here.” IDI 4 - facility nurse  **Women can see the results**  “M: What are your feelings about using RDT in routine ANC? R: It is nice because the provider who is seeing… the nurse seeing the client at the moment is able to test the client and get the results. So you are able to test by yourself and give the results.” IDI 8 - Nurse  **Low tech and easy to use, doesn’t require training**  “M: What do you think about the use of RDTs for diagnosis of malaria as an alternative to blood slides? R: I think it if more friendly. M: Mmm… R: It can be done at one spot and it can be done by the nursing aider, unlike the one for the microscopy that only requires the lab technologies. M: It is also quick. R: Yeah, it is also quick and can be used where there is no microscope.” IDI 8 – Nurse  **Could initially deter women from coming to ANC**  “R: Issues about pricking and withdrawing blood from clients always carry several issues at the community level so when the client gets to know that every time you go to the clinic they have to be pricked, before they come to accept the practice a lot of them can even pull out of the MCH. They do not like the pricks but with time they get used to it and it will be business as usual just like HIV now, it is normal that the first visit and after every three months you have to be pricked for HIV if you go to the clinic.” IDI 5 - facility nurse  **Drugs in ANC**  **One stop shop – the best way to serve women**  “M: My question is that how would you feel if the pregnant women attending ANC for testing who tested malaria positive were given their anti-malarial treatment by the ANC staff. R: I feel okay; when we talk of the hospital set up we have a reason as to why the MCH is always separated from all the departments. I think it should be under one roof; everything should be done under one roof. M: Yeah. R: So I feel okay because they are aware of the pharmacologist side. I do not have any problem when they dispense the drugs. M: Mmm. R: Yeah. So I think they should be served by the ANC here with the drugs without being sent to other departments.” IDI 13 - Lab technician  **One stop shop – ensures women get the treatment when they are found to be positive**  “R: It would be better because the waiting time is the main problem. If I see these clients and get through with them, it would be better for the client and we are sure they are given the quality services, because if I send her to the pharmacy, I don’t know whether she gets the drug or she has not got the drug. I wouldn’t be so sure this woman was treated of malaria, all I am sure of is that she had a positive blood sample.” IDI 3 - facility nurse  **One stop shop – reduce wait times for women, encourage them to attend ANC**  “R: Mmm, I think that would be convenient for the patient so. First it would avoid making the patients make a second queue at the pharmacy. You know within the clinic we have a system of first tracking them, then, if this client has been queuing here, and now you want him to see the clinical officer. They are not going to start queuing again. You make them go in because they had queued in the antenatal clinic. Yeah. M: Mmh. R: So that reduces the time that they spend in the clinic and by the end of the day, it will encourage mothers to come to the clinic.” IDI 5 - facility nurse  **ANC staff are the ones working with the women so they should dispense drugs**  “M: Ok thank you. And how would you feel if the pregnant women attending ANC who tested positive for malaria were given their anti malarial treatment by the ANC staff? R: Ok I would feel good because the ANC staffs are close to these pregnant mothers so they are the ones who are interacting with them.” IDI 2 - nurse  **ANC staff are not qualified, do not have necessary information to give out medications**  “R: Okay our strategy in this hospital or our policy in this hospital is that all drugs are dispensed at one particular place and that is the pharmacy. M: Mmmm… R: We did this because we realized that some of the staffs were not conversant with the prescription of the drugs and also they would just give drugs without explaining to the mother, the mother may not know the seriousness of the drug she is using.” IDI 9 - Sister in Charge  **Re-stocking is done through pharmacy so they need to oversee and dispense drugs**  “R:…As for as for the drug utilization, we give if the protocols are used because protocols of drug management says that any medicine that comes through this hospital should pass through the drug store and then from the drug store it is documented M: It must be… R: If protocol is to be observed, the pharmacist will use this to order it from the store then it comes here. When it has come here then nurse at the ANC will come here to get it, so if I’ve given 10 doses of Fansidar to her, then she comes after a week to get another 10 doses, I will know that she has given it there, in other words I can do reconsumption even if am not there giving from this angle…and then monitor.” IDI 18 - Pharmacist  Counselling  **Counselling may be needed for women to understand RDT for malaria**  “M: And the nature of the kit, because I understand that they are more less the same with HIV test kit, so what do you think about it, how can you tell me this is just for malaria and not for HIV/AIDS? R: (laughs) I will have to counsel you. At the beginning it would be a an issue because you know to accept this thing in our community is not easy, but through ongoing counselling she will have to accept and at the same time this mother would have passed through PMTC so she will know that this one is for malaria and this one for PMTC to test HIV. So there would be an ongoing counselling.” IDI 1 - facility nurse |
